# Seasonal Distribution and Meteorological Factors Associated with Hand, Foot, and Mouth Disease among Children in Xi’an, Northwestern China

**DOI:** 10.4269/ajtmh.19-0916

**Published:** 2020-03-09

**Authors:** Tianci Guo, Jifeng Liu, Junjiang Chen, Yao Bai, Yong Long, Baozhong Chen, Shuxuan Song, Zhongjun Shao, Kun Liu

**Affiliations:** 1 Department of Epidemiology, Ministry of Education Key Lab of Hazard Assessment and Control in Special Operational Environment, School of Public Health, Air Force Medical University, Xi’an, P. R. China;; 2 Department of Infectious Disease Control and Prevention, Xi’an Center for Disease Prevention and Control, Xi’an, P. R. China

## Abstract

Hand, foot, and mouth disease (HFMD) is a common infectious disease in the Asia-Pacific region that primarily affects children younger than 5 years. Previous studies have confirmed that the seasonal transmission of this disease is strongly related to meteorological factors, but the results are not consistent. In addition, the associations between weather conditions and HFMD in northwestern China have not been investigated. Therefore, we aimed to examine this issue in Xi’an, the largest city of northwestern China that has been suffering from serious HFMD epidemics. In the current study, data for HFMD and six meteorological factors were collected from 2009 to 2018. Using cross-correlation analysis, the Granger causality test, and the distributed lag nonlinear model, we estimated the quantitative relationships and exposure-lag–response effects between weekly meteorological factors and HFMD incidence among children. We found that the seasonal distribution of HFMD in Xi’an has two peaks each year and is significantly impacted by the weekly temperature, precipitation, and evaporation over an 8-week period. Higher values of temperature and evaporation had positive associations with disease transmission, whereas the association between precipitation and HFMD showed an inverted-U shape. The maximum relative risks (RRs) of HFMD for the weekly mean temperature (approximately 31.1°C), weekly cumulative evaporation (57.9 mm), and weekly cumulative precipitation (30.0 mm) were 1.56 (95% CI: 1.35–1.81), 1.40 (95% CI: 1.05–1.88), and 1.16 (95% CI: 1.11–1.70), respectively. The identified risk determinants and lag effects could provide important information for early interventions to reduce the local disease burden.

## INTRODUCTION

Hand, foot, and mouth disease (HFMD) is a common infectious disease caused by enteroviruses (e.g., EV-71, Coxsackievirus 16 [CV-A16], CV-A4, CV-A6, and CV-A10) that primarily affects children younger than 5 years.^1^ Although the symptoms are usually mild (fever, skin rash on the hands and feet, and vesicles in the mouth), some rare complications can lead to cognitive and motor disorders as well as death.^2,3^ Therefore, this disease poses a serious threat to public health in countries with a high incidence, such as China and other Asia-Pacific countries.^4–8^ China accounted for 86% of the total cases reported to the WHO between 2010 and 2014, and it reported an alarming number of approximately 2.7 million cases of HFMD, resulting in 384 deaths in 2014.^9^ Although vaccines against EV-71 for children aged 6 months to 5 years have been available since 2015, no effective vaccines for other Coxsackieviruses exist yet, and the incidence of HFMD has not decreased.^10^ Because HFMD is a major public health issue in China, ranking first among all notifiable infectious diseases and affecting more than two million children annually, it is of utmost importance to identify the risk factors of HFMD for better prevention and control of the disease.^4,11,12^

Some known risk factors for HFMD include hygiene, age, gender, and social contacts.^1^ In addition, HFMD is a climate-sensitive disease, and epidemics of HFMD have commonly shown seasonal variations and geographical differences.^1,4^ For example, HFMD has shown two peaks in May and October in southern China, one peak in June in northern China, and one peak in March or May in Singapore.^4,6^ Meteorological factors, such as temperature, relative humidity, precipitation, hours of sunshine, and air pressure, also have been shown to play important roles in transmission of this disease.^13,14^ However, associations between meteorological factors and HFMD have not been consistent in previous studies.^6,8,13,15,16^ A linear association has been reported between temperature and HFMD in Guangzhou (southern China),^13^ whereas studies from Shanghai, Singapore, and Japan have revealed nonlinear associations.^6,8,15^ Moreover, some meteorological factors, such as relative humidity and temperature, have shown opposite impacts on the transmission of HFMD in different regions.^13,16^ It has been hypothesized that these heterogeneous findings among studies are due to differences in types of data, modeling schemes, and, most importantly, region-specific characteristics, such as climatic, socioeconomic, demographic, and infrastructural conditions.^15,17^ Although a few studies have been conducted in China (Being, Shanghai, Guangzhou, Chengdu, and Hefei),^13,16,18–20^ to the best of our knowledge, no study has examined the association between meteorological factors and HFMD in northwestern China. This information is of utmost importance to design effective and targeted interventions to reduce the transmission of HFMD, according to local conditions.

Therefore, the aim of this study was to assess the association between weather conditions and HFMD in Xi’an, the largest city in northwestern China with serious HFMD epidemics. Specifically, we aimed to 1) explore the incidence of HFMD in Xi’an; 2) assess the association between epidemiological characteristics, especially the seasonal distribution, and incidence of HFMD; and 3) evaluate the exposure-lag–response effects of meteorological factors on the incidence of HFMD.

## MATERIALS AND METHODS

### Study area.

Xi’an lies on the Wei River Basin, a flood plain created by eight surrounding rivers and streams in the central part of Shaanxi Province with a high-density population, where it is suitable for the spread of HFMD. From 2008 to 2015, a total of 154,869 HFMD cases were reported in Xi’an city, with an annual incidence of 235.01 per 100,000 people, which is nearly two times the national average incidence during the same period.^21^ Xi’an consists of 13 counties and districts with an administrative area of 9,983 km^2^, and the total population was more than 10 million residents in 2018 (Supplemental Figure 1). The city has an average elevation of 400 m above sea level and a typical temperate continental monsoon climate, with pleasant temperatures, moderate rainfall, and four distinct seasons: hot and wet in the summer, dry and seldom snowy in the winter, and prone to extended periods of rain in the spring and autumn. January is the coldest month, with an average temperature of −1.3°C; and July is the hottest month, with an average temperature of 26.7°C. The average annual rainfall is 604.2 mm, which is concentrated in July, August, and September (available from https://www.tianqi.com/xian/).

### Data source.

Case records of HFMD in Xi’an city from January 1, 2009 to December 31, 2018 were obtained from the Xi’an Center for CDC. We included all cases, without any exclusion criteria. In China, all clinics are obliged to report HFMD cases to the local CDC within 24 hours.^22^ The HFMD cases were diagnosed according to the guidelines provided by the National Health and Family Planning Commission of the People’s Republic of China (version 2008).^23^ Information regarding gender, age, onset date of symptoms, and residential address was collected for each case from the case records. The annual demographic data during the study period were obtained from the Xi’an Bureau of Statistics. A digital township-level map of Xi’an city was gathered from the China Resource and Environment Data Cloud Platform (http://www.resdc.cn/). Data on six local meteorological variables—temperature, precipitation, evaporation, atmospheric pressure, relative humidity, and sunshine duration—were collected daily during the study period from the Chinese Bureau of Meteorology (http://data.cma.cn/).

### Statistical analysis.

To display the epidemiological characteristics of HFMD in Xi’an in a clear manner, a bar chart of annual HFMD cases and average age- and gender-specific incidences was created for the study period, and a spatial distribution map of the annual HFMD incidence was produced at the township level. Because HFMD cases involving children younger than 5 years accounted for most of the total cases, we counted the weekly number of reported HFMD cases in children younger than 5 years and calculated the weekly incidence in Xi’an, which was expressed by the seasonal distribution of meteorological factors and HFMD incidence using time series plots. Then, we used Spearman’s correlation analysis to calculate preliminary correlations among meteorological factors and the HFMD incidence. In addition, a Granger causality test was performed for each meteorological factor to determine the potential effect of meteorological variability on the transmission of HFMD. Subsequently, the variables selected in the Granger causality test were included in a distributed lag nonlinear model (DLNM) to examine the potentially nonlinear and delayed effects of meteorological factors on disease transmission. The DLNM is based on the definition of cross-basis, a bidimensional function expressed as the combination of two basic functions, which depicts the effects of predictor and lag simultaneously. Two functions, “crossbasis” and “crosspred,” are included in the DLNM and are used to study the lag effect and prediction, respectively, as well as to estimate the exposure–response prediction across the maximum lag period.^24^ In the current study, we used the DLNM combined with the generalized additive model to analyze the effects. A number of covariates were incorporated through the Poisson regression model, as follows:

Yt∼Poisson(μ)=α+NS(β, df, lag, df) +NS(time, df)+week

where Yt is the incidence of HFMD on week *t*, α is the intercept, NS is a natural cubic spline used to model the nonlinear relationship between the meteorological variables and the incidence, β is the examined meteorological variable that was strongly related to the incidence, df is the degrees of freedom of each meteorological variable per year, time is the indicator variable used to control long-term trends and seasonality, and week is an ordinal variable for the week of each year.

The analysis was performed using R software version 3.6.1 with the “lmtest” and “dlnm” packages. All statistical tests were two sided, and a *P*-value < 0.05 was considered statistically significant.

## RESULTS

### Epidemiological characteristics of HFMD and meteorological factors.

**Figure 1.**
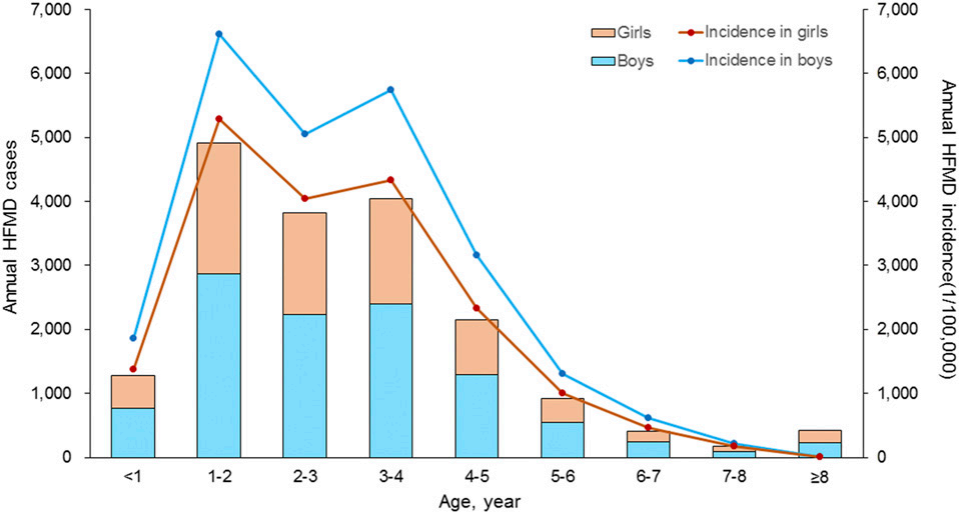
Age and gender distribution of hand, foot, and mouth disease cases in Xi’an city, northwestern China, 2009–2018. This figure appears in color at www.ajtmh.org.

**Figure 2.**
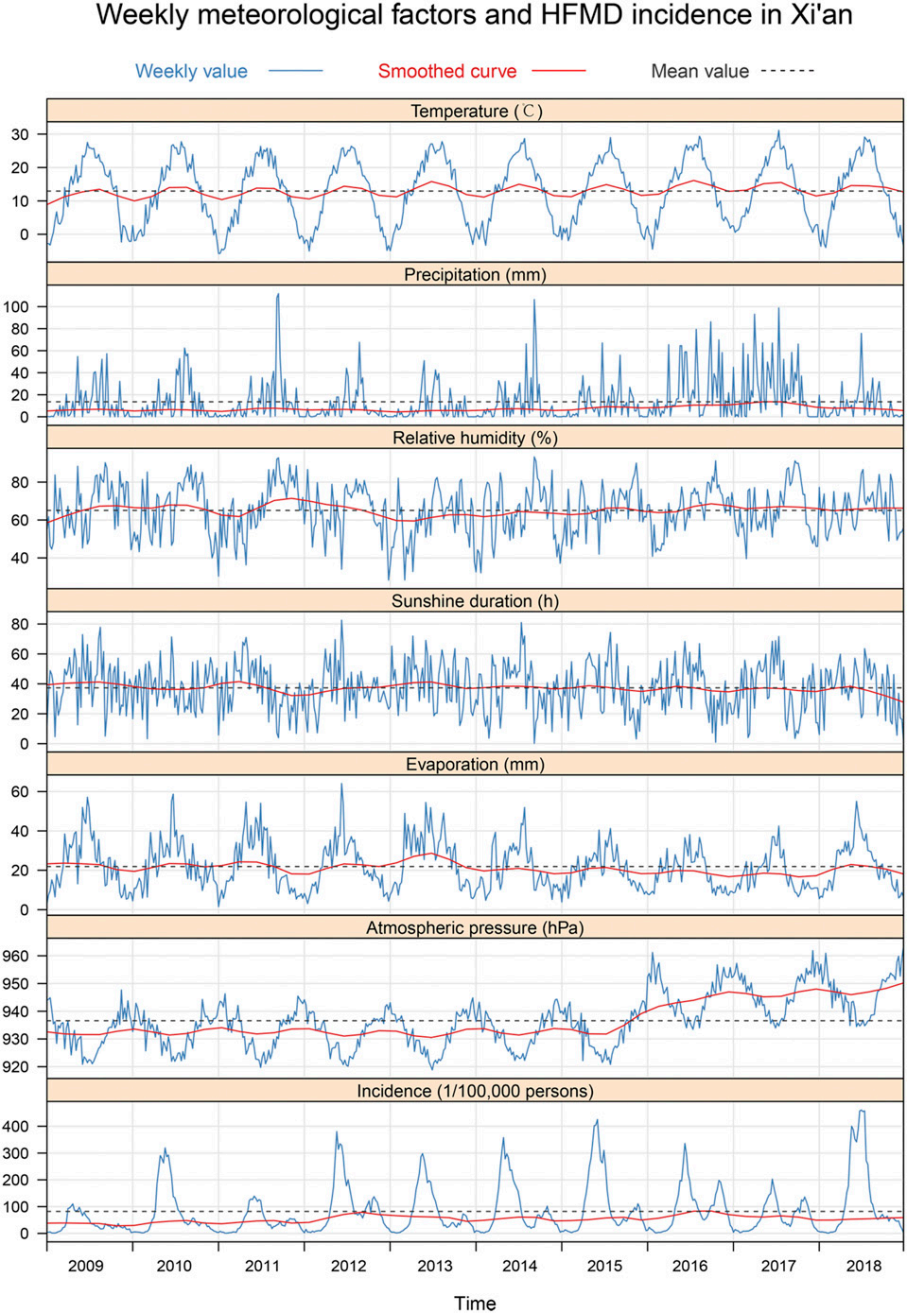
Weekly incidence of hand, foot, and mouth disease (HFMD) and meteorological variables in Xi’an city, northwestern China, 2009–2018. (From top to bottom, the curves are temperature, precipitation, relative humidity, sunshine duration, evaporation, atmospheric pressure, and incidence of HFMD, respectively). This figure appears in color at www.ajtmh.org.

From January 1, 2009 to December 31, 2018, a total of 181,358 cases and 18 deaths due to HFMD were reported in Xi’an. The ages of the patients ranged from 1 day to 78 years (median: 2 years), and the mean (SD) age was 2.55 (2.51) years. Children younger than 5 years with HFMD accounted for 94.5% (162,152/181,358) of the total cases. The ratio of males to females of all cases was 1.43:1, and males had a significantly higher incidence than females among all age-groups (*P* < 0.001). The highest annual age-specific incidence occurred between 1 and 4 years of age, with an incidence greater than 5,000/100,000 people (Figure 1). The high epidemic region of HFMD mainly occurred at the junctions of urban–rural zones around Xi’an (Supplemental Figure 2). Details of the weekly meteorological factors and HFMD incidence during the study period are shown in Table 1, Figure 2. Specifically, a weekly time-series diagram of HFMD incidence in children younger than 5 years and meteorological factors indicated significant seasonal variation (Figure 2). The HFMD incidence had a slow increasing trend, showing an obvious bimodal curve with a large peak in late spring and early summer from the 17th week to the 29th week of each year, and a small peak in late autumn and early winter between the 38th week and the 49th week (Figure 2). The weekly values for average temperature, accumulative precipitation, accumulative evaporation, average atmospheric pressure, average relative humidity, and accumulative sunshine duration are shown in Table 1.

**Table 1:** Descriptive statistics of weekly meteorological factors and HFMD incidence among children younger than 5 years in Xi’an, northwestern China, 2009–2018

Variable	Min	P_25_	P_50_	P_75_	Max	Mean ± SD
Temperature (°C)	−5.75	3.99	13.94	21.10	31.13	12.94 ± 9.58
Precipitation (mm)	0	0.52	6.16	18.40	111.79	13.58 ± 19.00
Evaporation (mm)	1.54	11.92	20.01	29.84	64.09	21.89 ± 11.88
Atmospheric pressure (hPa)	918.82	929.54	936.31	942.35	963.28	936.63 ± 9.41
Relative humidity (%)	28.31	55.28	65.72	74.82	93.29	65.06 ± 13.02
Sunshine duration (h)	0.23	24.89	37.40	49.00	82.54	37.33 ± 16.24
HFMD incidence (1/100,000 people)	0.74	17.94	48.13	109.44	460.45	82.08 ± 93.84

HFMD = hand, foot, and mouth disease; Max = the maximum level of the variable; Min = minimum level of the variable; P_25_ = the 25th percentile of the variable; P_50_ = the 50th percentile of the variable; P_75_ = the 75th percentile of the variable.

**Figure 3.**
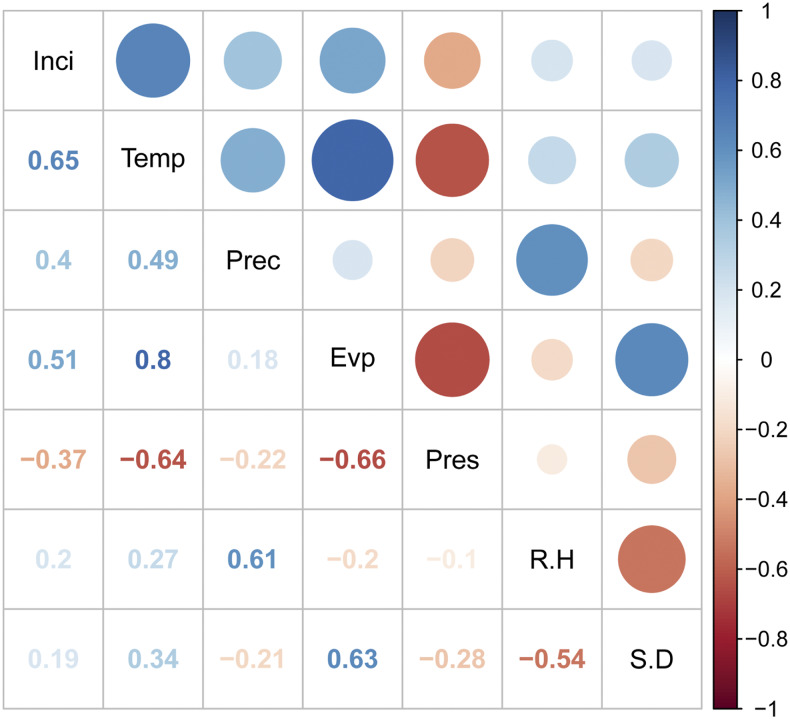
Cross-correlation coefficients between the weekly meteorological variables and the incidence of hand, foot, and mouth disease (HFMD) in Xi’an city, northwestern China, 2009–2018 (Inci: weekly incidence of HFMD; Temp: weekly mean temperature; Prec: weekly cumulative precipitation; Evp: weekly cumulative evaporation; Pres: weekly mean atmospheric pressure; R.H: weekly mean relative humidity; S.D: weekly cumulative sunshine duration). This figure appears in color at www.ajtmh.org.

The cross-correlation analysis revealed that three meteorological factors—temperature, precipitation, and evaporation—were positively correlated with the HFMD incidence, and atmospheric pressure was negatively associated with the disease (Figure 3). The results of the Granger causality tests indicated that the temporal distribution of weekly HFMD incidence was significantly affected by temperature, precipitation, and evaporation (*P* < 0.05) (Table 2).

**Table 2:** Granger causality tests for each meteorological variable and the weekly incidence of hand, foot, and mouth disease in Xi’an city, northwestern China, 2009–2018

	Temperature	Precipitation	Relative humidity	Sunshine duration	Evaporation	Atmospheric pressure
*F*-statistic	3.808	1.996	1.353	1.297	2.469	1.041
*P*-value	< 0.001	0.0451	0.215	0.257	0.012	0.404

### Nonlinear and lag effects of meteorological factors on the HFMD incidence.

**Figure 4.**
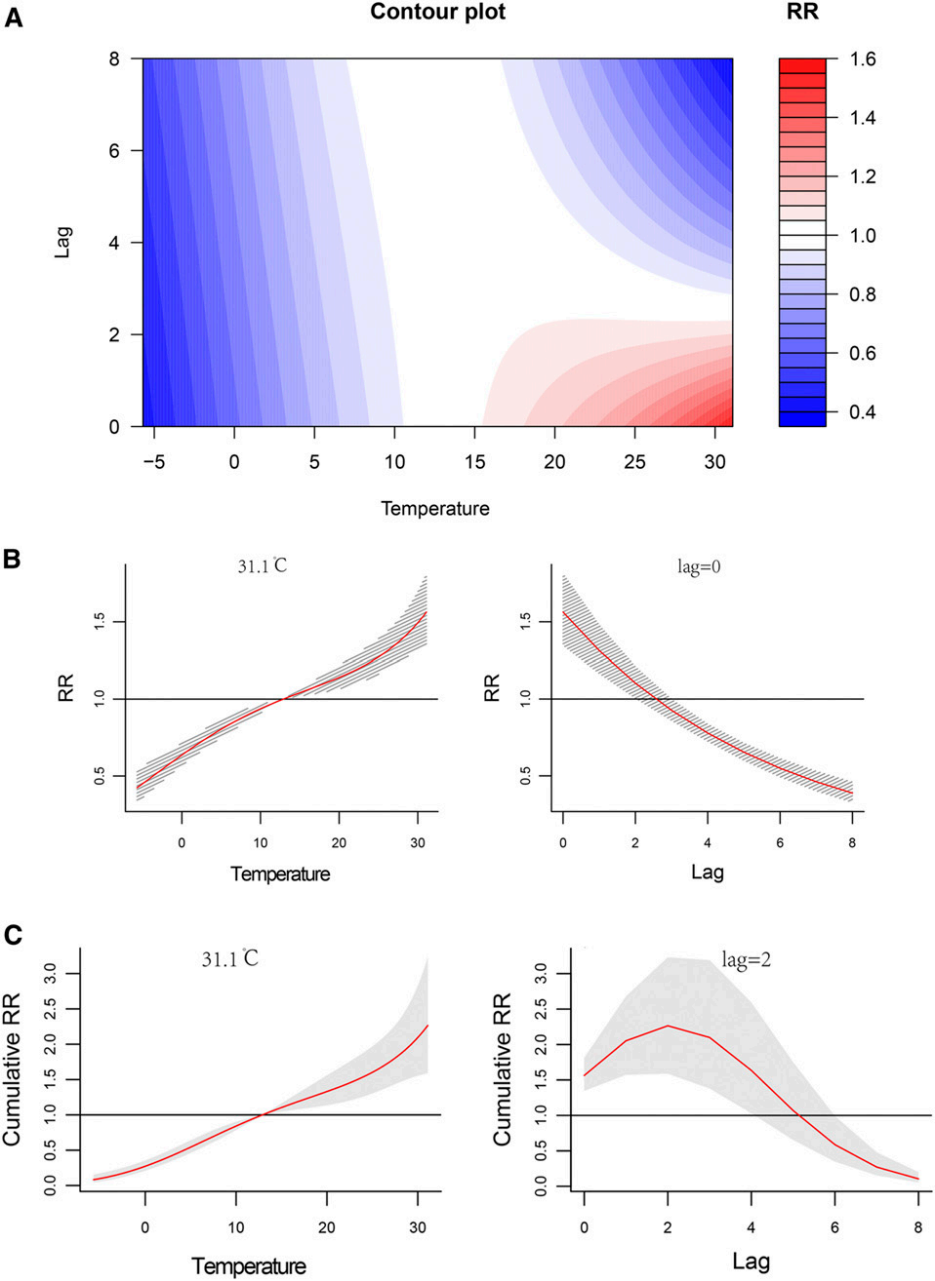
Nonlinear and lag effects of temperature on the incidence of hand, foot, and mouth disease (HFMD) in Xi’an city, northwestern China, 2009–2018. (**A**) Contour plots of the combined effect of lag time (weeks) and temperature on the relative risk (RR) of HFMD transmission. (**B**) Effects of specific temperature and lag time (weeks) on the RR of HFMD transmission. The red lines indicate the mean RRs, and the gray lines indicate the 95% CI. (**C**) Effect of specific temperature and lag time (weeks) on the cumulative risk of HFMD transmission. The red lines indicate the mean cumulative risks, and the gray areas correspond to the 95% CIs. This figure appears in color at www.ajtmh.org.

Figure 4A shows the comprehensive association between the average weekly temperature and the incidence of HFMD along with the lag time (0–8 weeks) (also shown in Supplemental Figure 3). Within 2 weeks, the relative risk (RR) of HFMD increased significantly as the temperature increased, but after 2 weeks, the impact of temperature became negative. The separate effects on the RRs together with the 95% CI are shown in Figure 4B. The RR of HFMD with temperature was positive at a lag time of 0, and it decreased substantially as the lag time increased. The maximum RR was 1.56 (95% CI: 1.35–1.81) at approximately 31.1°C in the current week (lag time of 0) (Table 3). The effect of temperature on the cumulative risk of HFMD is shown in Figure 4C. A higher temperature increased the cumulative risk from a lag time of 0 to 2 weeks; and the highest RR value of 2.27 (95% CI: 1.59–3.23) appeared at approximately 31.1°C, with a lag time of 2 weeks.

**Table 3:** Separate and cumulative effects of meteorological factors on the weekly hand, foot, and mouth disease incidence and the corresponding variable values in Xi’an city, northwestern China, 2009–2018

Variable	Separate effect			Cumulative effect		
	Maximum RR (95% CI)	Variable value	Lag time (weeks)	Maximum RR (95% CI)	Variable value	Lag time (weeks)
Temperature (°C)	1.56 (1.35–1.81)	31.1	0	2.27 (1.59–3.23)	31.1	2
Precipitation (mm)	1.16 (1.11–1.70)	30.0	0	2.03 (1.73–2.37)	24.9	7
Evaporation (mm)	1.40 (1.05–1.88)	57.9	0	2.40 (1.66–3.46)	41.7	5

RR = relative risk.

**Figure 5.**
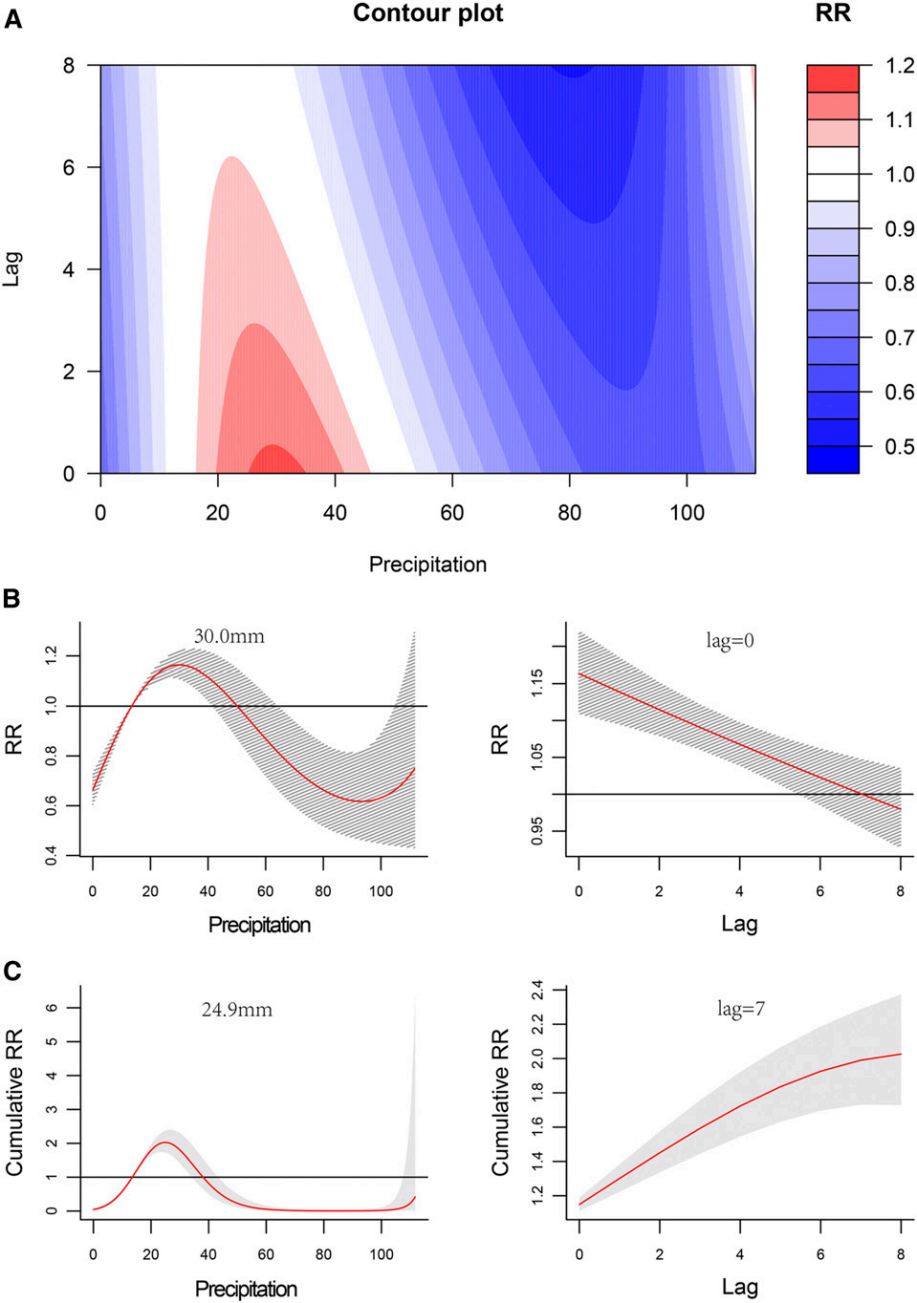
Nonlinear and lag effects of precipitation on the incidence of hand, foot, and mouth disease (HFMD) in Xi’an city, northwestern China, 2009–2018. (**A**) Contour plots of the combined effect of lag time (weeks) and precipitation on the relative risk (RR) of HFMD transmission. (**B**) Effects of specific precipitation and lag time (weeks) on the RR of HFMD transmission. The red lines indicate the mean RRs, and the gray lines indicate the 95% CIs. (**C**) Effects of specific precipitation and lag time (weeks) on the cumulative risk of HFMD transmission. The red lines indicate the mean cumulative risks, and the gray areas indicate the 95% CIs. This figure appears in color at www.ajtmh.org

The association between weekly precipitation and HFMD showed an inverted-U shape during the 8-week period following the measurement. With an increase of precipitation, the RR of HFMD rose to the peak at about 30 mm of precipitation and then declined rapidly (Figure 5A, Supplemental Figure 4). With a weekly precipitation of 20–40 mm, the separate and cumulative effects were positive (Figure 5B and C). The separate effects were the strongest at about 30.0 mm of precipitation with a lag time of 0 weeks, with a maximum RR value of 1.16 (95% CI: 1.11–1.70) (Table 3); and the largest cumulative effects occurred at 24.9 mm of precipitation with a lag time of 7 weeks, with a RR value of 2.03 (95% CI: 1.73–2.37) (Table 3).

**Figure 6.**
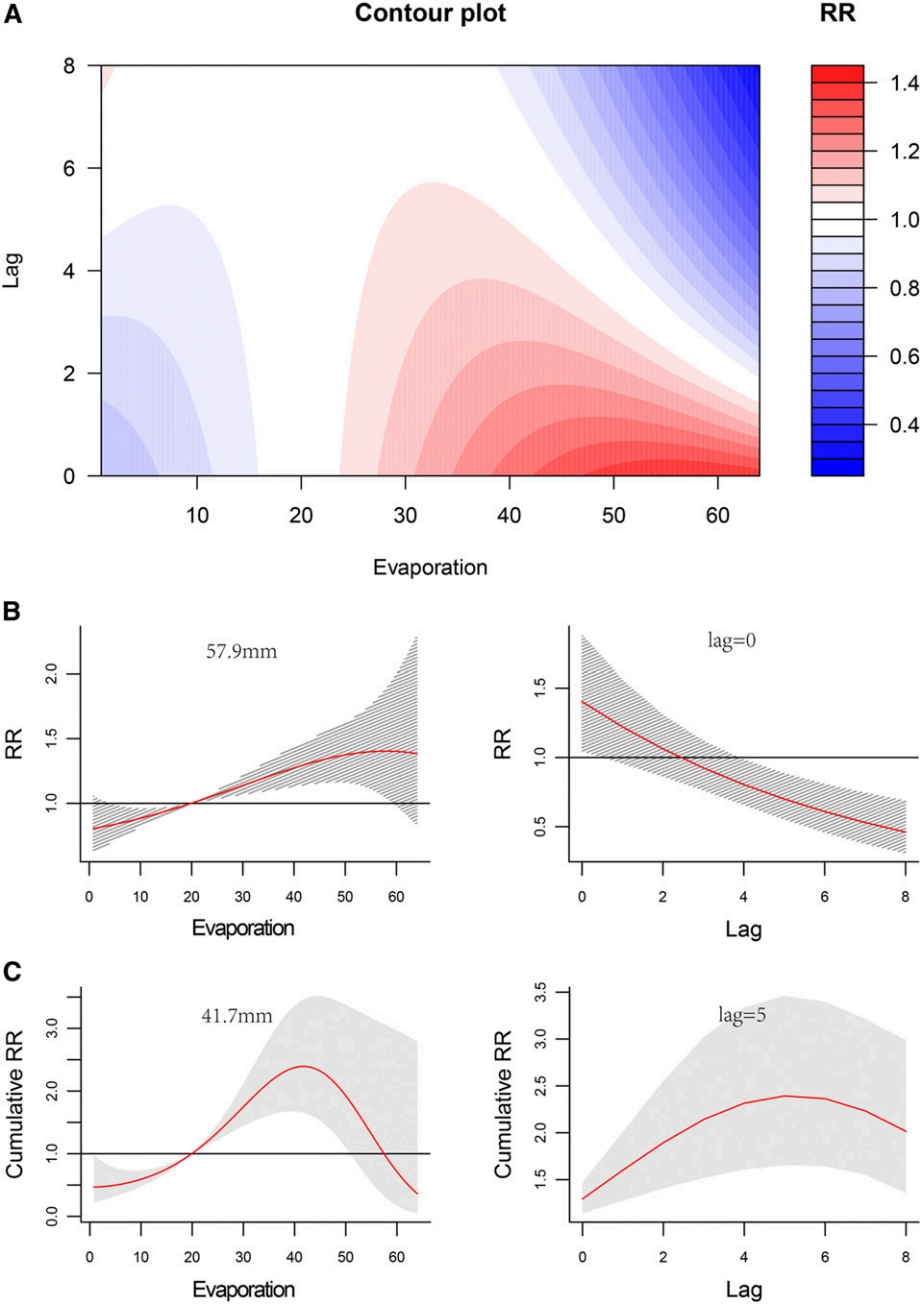
Nonlinear and lag effects of evaporation on the incidence of hand, foot, and mouth disease (HFMD) in Xi’an City, northwestern China, 2009–2018. (**A**) Contour plots of the combined effect of evaporation and lag time (weeks) on the relative risk (RR) of HFMD transmission. (**B**) Effects of specific evaporation and lag time (weeks) on the RR of HFMD transmission. The red lines indicate the mean RRs, and the gray lines indicate the 95% CIs. (**C**) Effect of specific evaporation and lag time (weeks) on the cumulative risk of HFMD transmission. The red lines indicate the mean cumulative risks, and the gray areas indicate 95% CIs. This figure appears in color at www.ajtmh.org

Figure 6A shows the combined effect of lag time and evaporation on the RR of HFMD. Given the high correlation between evaporation and temperature (*r* = 0.80), the effects of weekly evaporation on HFMD were similar to those of temperature; however, the effects of evaporation lasted a longer time (5 weeks), with higher values between 30 mm and 40 mm of evaporation (Supplemental Figure 5). The separate RR increased with a rise of evaporation, and the coefficient of variation increased simultaneously. The RR of weekly evaporation of 57.9 mm with a lag time of 0 week was the highest, with a value of 1.40 (95% CI: 1.05–1.88) (Figure 6B, Table 3). The largest cumulative RR value of 2.40 (95% CI: 1.66–3.46) appeared at an evaporation of approximately 41.7 mm, with a lag time of 5 weeks (Figure 6C, Table 3).

## DISCUSSION

In the current study, a total of 181,358 HFMD cases were reported in Xi’an city, with an annual incidence of 213.88 per 100,000 people, ranking first among all notifiable infectious diseases from 2009 to 2018. We found that children younger than 5 years accounted for 94.5% of the HFMD cases, and males had a higher incidence than females. Our results demonstrated that the weekly meteorological variables, especially temperature, precipitation, and evaporation, contributed significantly to the seasonal fluctuations of HFMD incidence among children. Significantly, we observed nonlinear lag effects in the relationships between short-term weather conditions and HFMD, thus providing valuable evidence for decisions regarding local public health. To the best of our knowledge, this is the first study to estimate the seasonal distribution and risk determinants of HFMD among children in Xi’an, northwestern China, in a systematic manner.

The incidence of HFMD observed in the current study was significantly higher than the average national incidence as well as that for other large cities in China (such as Beijing, Wuhan, Taiyuan, Shenyang, and Tianjin).^21,25–28^ The results of previous studies are consistent with the current finding that children younger than 5 years account for most of the HFMD cases, and males have a higher incidence than females.^29^ However, prior studies have found different seasonal patterns of HFMD compared with the current study. In the present study, we found a large peak for HFMD incidence in late spring and early summer as well as a small peak in late autumn and early winter in Xi’an; this finding was different from those observed in northern China, which only showed one peak in June.^4^ The observed heterogeneity supported the hypothesis that region-specific characteristics could explain the discrepant findings among studies and reinforced the importance of investigating the region-specific epidemiology of HFMD for more tailored intervention and prevention policies.^15,17^

In addition, the current study used the DLNM to describe the potentially nonlinear and lag effects in the time-series data; this model has been widely used to estimate the relationships between seasonal variations of infectious diseases, such as malaria, HFMD, tuberculosis, and mumps, and meteorological factors.^15,16,19,30–32^ As a result, we found that meteorological factors, including temperature, precipitation, and evaporation, had a significant impact on the occurrence of HFMD, with a lag period of 0 to 8 weeks. The positive role that temperature plays in the incidence of HFMD is well understood. The increased temperature may lead to enhanced survival, reproduction, and transmission of enteroviruses as well as higher chances of exposure for children through contact.^33–35^ An increased temperature also has been shown to lower immunity in humans as well as to facilitate the spread and susceptibility of HFMD among children.^36–38^ In the present study, we observed a positive impact of temperature within a short time of 2 weeks, after which the impact became negative. This may be due to a rapid change in temperature, which may cause people to alter their activities accordingly. Evaporation has been observed to have a high correlation with temperature; therefore, not surprisingly, the effects of evaporation on HFMD were similar to those of temperature. Furthermore, higher levels of evaporation may increase the transmission of virus through the additional promotion of air flow.^39^ Global climate change may be a primary contributor to the change in temperature, and children represent the most vulnerable group and are particularly at greater risk of disease.^40^

Previous studies have found a negative linear relationship between monthly precipitation and HFMD in eastern central China and Vietnam, which is different from the inverted-U pattern relationship observed in Xi’an.^41–44^ The positive association between precipitation and HFMD may be due to the moisture content in the surface soil or water, which mimics the water content in human feces, thus enabling and activating the virus and affecting the activity and transmission of the enteroviruses so that they are more contagious and survive longer.^45,46^ On the other hand, the negative association between precipitation and HFMD could be explained by the fact that because of excessive precipitation, children spend less time participating in outdoor activities and have a decreased risk of coming into contact with enteroviruses. Thus, the incidence of HFMD decreases with increasing precipitation.

Over the past few decades, HFMD has emerged as a serious public health threat worldwide because of its high incidence and severe complications.^1^ Although the incidence of HFMD in Xi’an is high and has shown a slowly increasing trend, the case fatality rate (CFR) of this disease has declined and is significantly lower than the national average level.^47^ The increased incidence and decreased CFR may reflect the improvements in disease reporting and surveillance, suggesting that HFMD cases have been reported in a timely and accurate manner, subsequently leading to timely and effective treatment. Although the incidence of HFMD in Xi’an has not decreased after the widespread use of the EV-71 vaccine since 2017, the number of severe HFMD cases due to EV-71 infection has decreased dramatically, which is a good sign. However, high and inapparent infection rates of HFMD have been observed mainly among children younger than 4 years, peaking between April and June in Xi’an, suggesting that preventative measures should be installed for high-risk children in addition to vaccination before the epidemic seasons every year.^48^

The results from the current study have important public health implications, such as meteorological factors, namely, weekly temperature, precipitation, and evaporation, could be considered as useful references in the early warning system for the disease transmission. In Xi’an, high temperature and evaporation days (weekly temperature higher than 20°C and weekly evaporation higher than 30 mm), and moderate precipitation days (weekly precipitation of 20–40 mm) would significantly raise the risk of HFMD. Therefore, targeted strategies and measures to prevent and control epidemics should be conducted in this period, such as rigorous disinfection measures in the kindergartens and playgrounds, and public health education regarding combating HFMD could be enhanced, through TV, radio, or posters, to increase the awareness of HFMD among people.

Several limitations merit consideration. First, we used data from a hospital-based passive surveillance system, which only captured patients with HFMD who sought medical care, and did not include all cases. Therefore, the prevalence of HFMD in the current study may be underestimated. Second, some information was not collected in the hospital system, such as the types of pathogens responsible for the HFMD cases; thus, we were unable to investigate the specific impacts of meteorological factors on different pathogens, which may have different tolerances to meteorological factors, such as temperature and humidity. In addition, we could not explore the residual confounding from demographic, socioeconomic, behavioral, and physiological factors that may explain the associations between meteorological variables and HFMD incidence. Although the current data source was from the governmental sector and is likely to be highly credible, data were not available to demonstrate that the accuracy of the reporting system is the same in all areas in this study. Future studies with a prospective study design and comprehensive data collection in Xi’an and northwestern China are warranted to validate our findings and address the aforementioned issues.

## CONCLUSION

Our study showed significant associations between meteorological factors (temperature, precipitation, and evaporation) and seasonal variation of HFMD in Xi’an, China. The key determinants of HFMD transmission and the identified lag effects in the current study provide significant evidence and useful references for early warning and the development of targeted strategies and measures to prevent and control local epidemics more effectively.

## Supplemental figures

Supplemental materials
