# Supplementary material for: Seasonal Distribution and Meteorological Factors Associated with Hand, Foot, and Mouth Disease among Children in Xi’an, Northwestern China
Source: Am J Trop Med Hyg. 2020 Mar 9;102(6):1253–62. doi: 10.4269/ajtmh.19-0916 (PMC7253124; doi:10.4269/ajtmh.19-0916)
Supplement: Supplementary file 1 [file tpmd190916.SD1.docx]

**Supplemental Figure Legends:**

**Supplemental Figure S1. Study site in China.** The map was created by Kun Liu in ArcGIS 10.1 Software, ESRI Inc., Redlands, CA, USA, (<https://www.arcgis.com/index.html>).

**Supplemental Figure S2. Annual incidence of HFMD in Xi’an City at the township level, 2009-2018.** The map was created by Kun Liu in ArcGIS 10.1 Software, ESRI Inc., Redlands, CA, USA, (<https://www.arcgis.com/index.html>).

**Supplemental Figure S3. Three-dimensional graph of the relationship between weekly average temperature and the incidence of HFMD in 8 weeks.**

**Supplemental Figure S4. Three-dimensional graph of the relationship between weekly cumulative precipitation and the incidence of HFMD in 8 weeks.**

**Supplemental Figure S5. Three-dimensional graph of the relationship between weekly cumulative evaporation and the incidence of HFMD in 8 weeks.**
