# Supplementary figures and images for: Seasonal Distribution and Meteorological Factors Associated with Hand, Foot, and Mouth Disease among Children in Xi’an, Northwestern China
Source: Am J Trop Med Hyg. 2020 Mar 9;102(6):1253–62. doi: 10.4269/ajtmh.19-0916 (PMC7253124; doi:10.4269/ajtmh.19-0916)

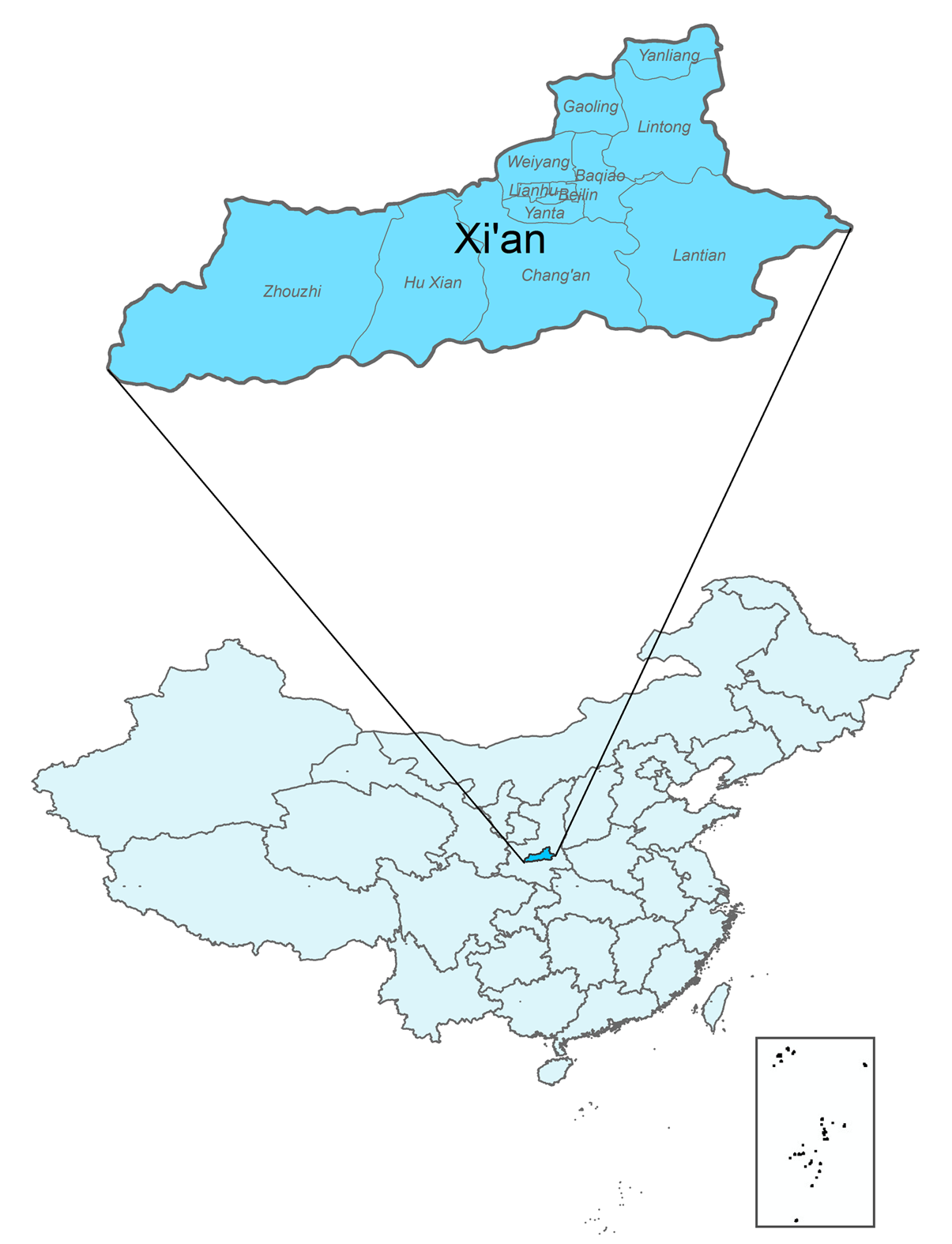

Supplement: Supplementary file 2 [file tpmd190916.SF1.tif]

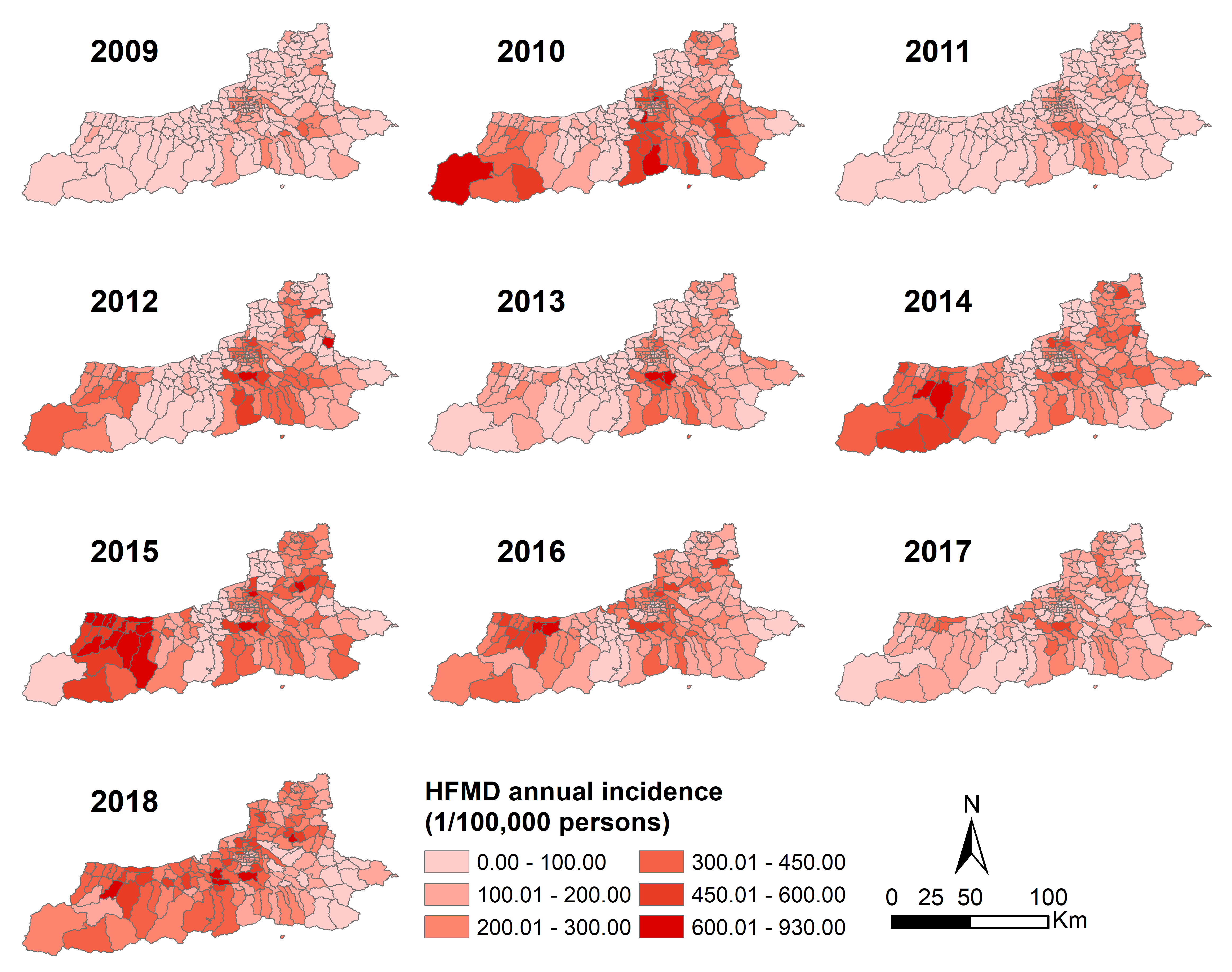

Supplement: Supplementary file 3 [file tpmd190916.SF2.tif]

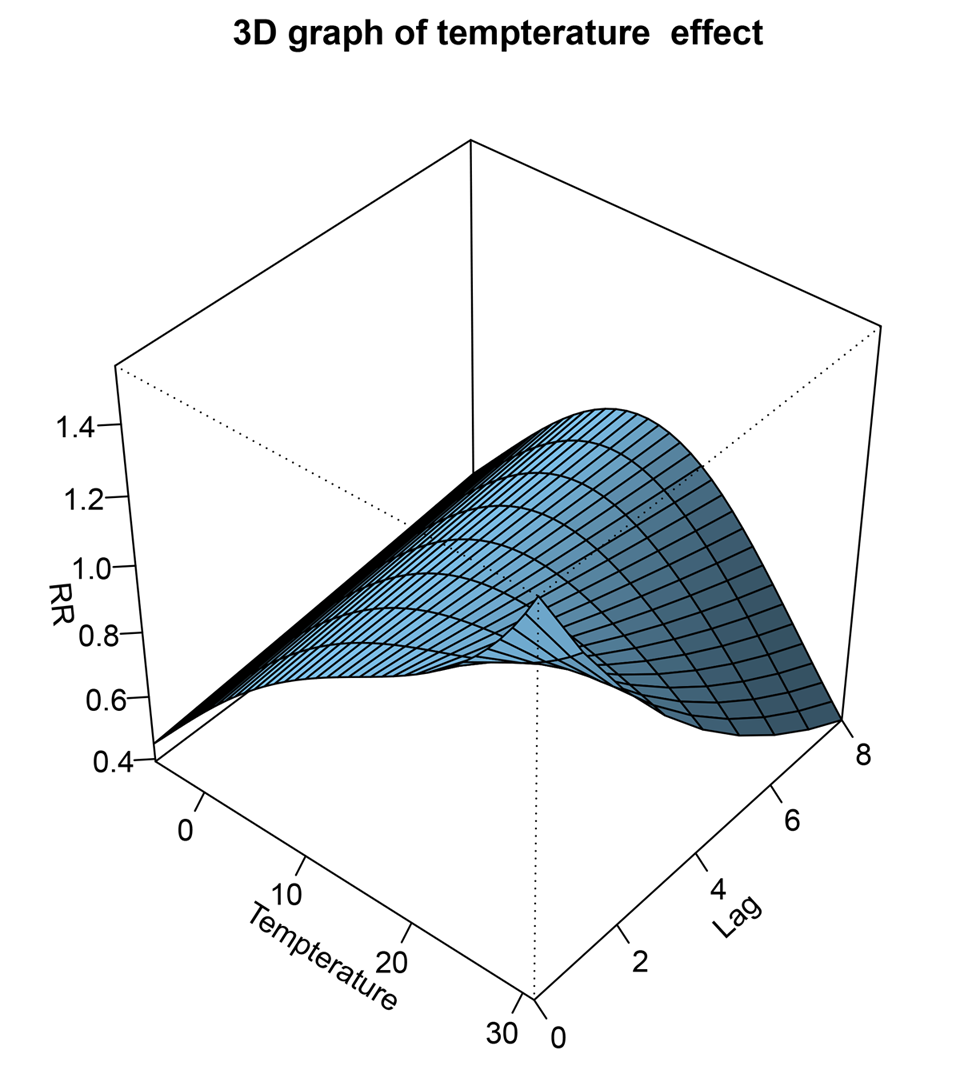

Supplement: Supplementary file 4 [file tpmd190916.SF3.tif]

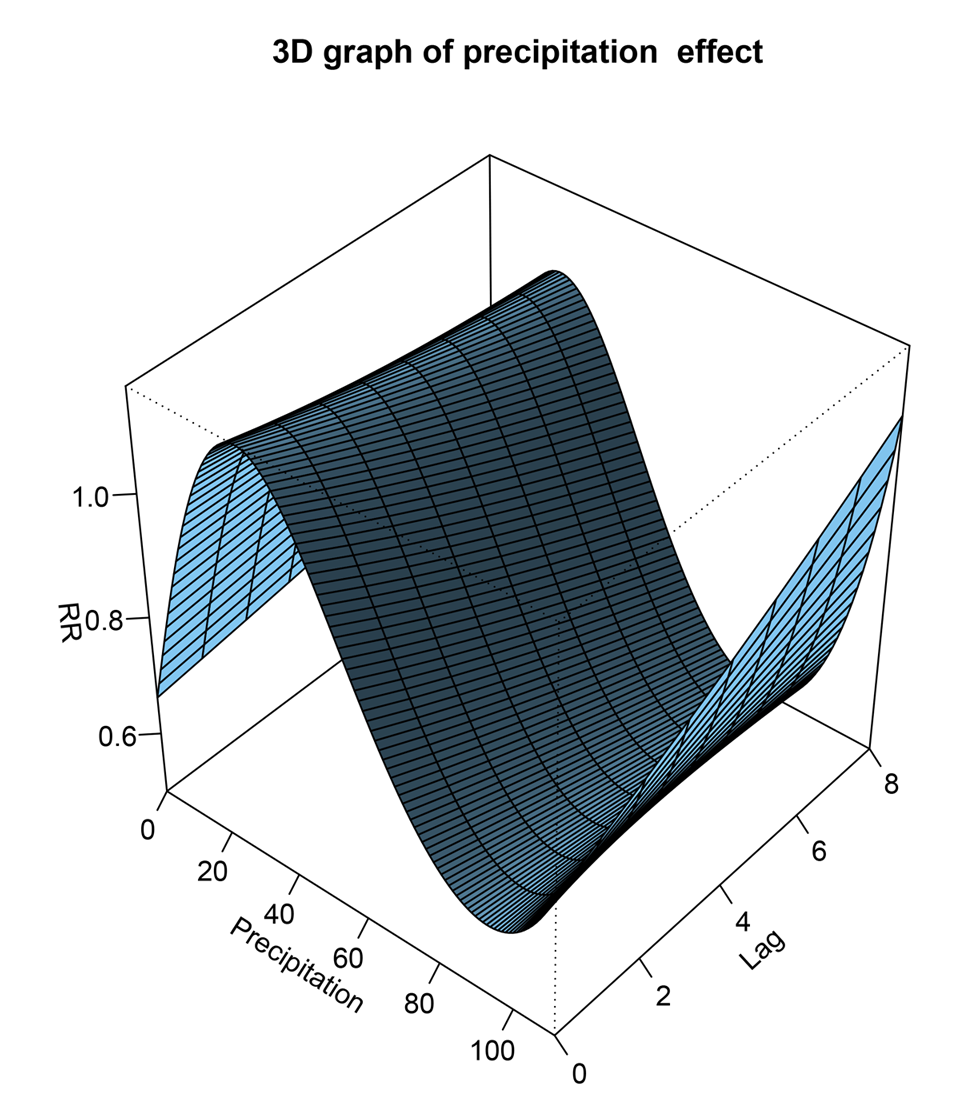

Supplement: Supplementary file 5 [file tpmd190916.SF4.tif]

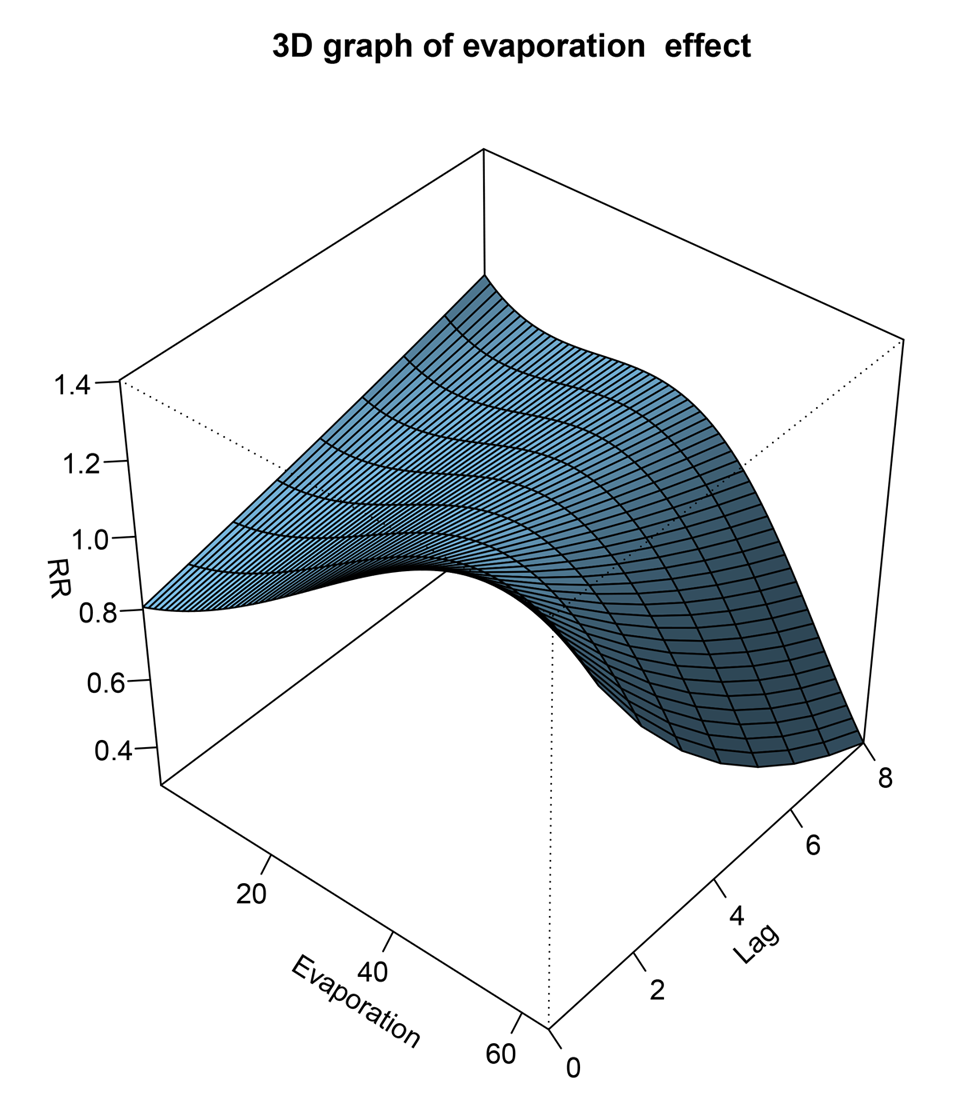

Supplement: Supplementary file 6 [file tpmd190916.SF5.tif]
